# Emerging Evidence of Translational Control by AU-Rich Element-Binding Proteins

**DOI:** 10.3389/fgene.2019.00332

**Published:** 2019-05-02

**Authors:** Hiroshi Otsuka, Akira Fukao, Yoshinori Funakami, Kent E. Duncan, Toshinobu Fujiwara

**Affiliations:** ^1^ Graduate School of Frontier Sciences, University of Tokyo, Kashiwa, Japan; ^2^ Kindai University, Higashi-osaka, Japan; ^3^ University Medical Center Hamburg-Eppendorf, Hamburg, Germany

**Keywords:** RNA-binding proteins, AU-rich element, ARE-binding proteins, translational control, mRNA decay

## Abstract

RNA-binding proteins (RBPs) are key regulators of posttranscriptional gene expression and control many important biological processes including cell proliferation, development, and differentiation. RBPs bind specific motifs in their target mRNAs and regulate mRNA fate at many steps. The AU-rich element (ARE) is one of the major cis-regulatory elements in the 3′ untranslated region (UTR) of labile mRNAs. Many of these encode factors requiring very tight regulation, such as inflammatory cytokines and growth factors. Disruption in the control of these factors’ expression can cause autoimmune diseases, developmental disorders, or cancers. Therefore, these mRNAs are strictly regulated by various RBPs, particularly ARE-binding proteins (ARE-BPs). To regulate mRNA metabolism, ARE-BPs bind target mRNAs and affect some factors on mRNAs directly, or recruit effectors, such as mRNA decay machinery and protein kinases to target mRNAs. Importantly, some ARE-BPs have stabilizing roles, whereas others are destabilizing, and ARE-BPs appear to compete with each other when binding to target mRNAs. The function of specific ARE-BPs is modulated by posttranslational modifications (PTMs) including methylation and phosphorylation, thereby providing a means for cellular signaling pathways to regulate stability of specific target mRNAs. In this review, we summarize recent studies which have revealed detailed molecular mechanisms of ARE-BP-mediated regulation of gene expression and also report on the importance of ARE-BP function in specific physiological contexts and how this relates to disease. We also propose an mRNP regulatory network based on competition between stabilizing ARE-BPs and destabilizing ARE-BPs.

## Introduction

**Figure 1:**
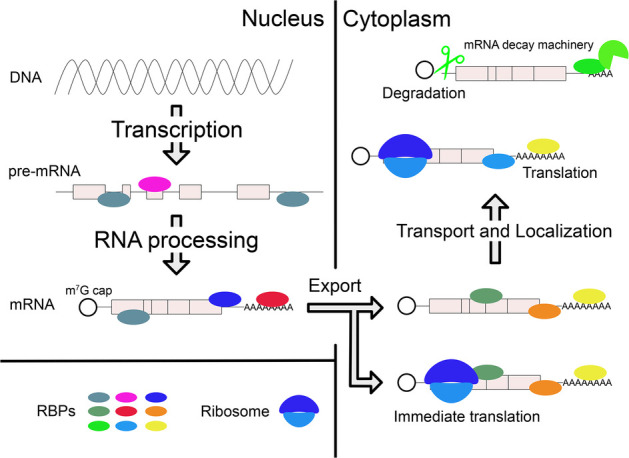
Posttranscriptional regulations of gene expression by RBPs. After transcription, RBPs bind pre-mRNA and regulate RNA processing in the nucleus. Mature mRNA is transported to cytoplasm by other RBPs. In the cytoplasm, various RBPs control the different mRNA fates, which include localization, translation, and degradation. Collectively, these effects achieve proper gene expression within specific cell types and in response to specific biological regulatory signals. They can also lead to pathological conditions when regulation is compromised, for example, due to mutations in the gene encoding a specific RBP.

Transcribed pre-mRNAs are subject to RNA processing in the nucleus, such as capping, polyadenylation, and splicing. Subsequently, processed mRNAs are exported to the cytoplasm (Bjork and Wieslander, 2017). In some cases, mRNAs are immediately translated, but they can also be transported to various subcellular compartments prior to translation. mRNAs are also turned over in the cytoplasm through regulated decay (Garneau et al., 2007). All of these posttranscriptional regulatory steps are important for proper gene expression and are themselves highly regulated. Interaction between RNA-binding proteins (RBPs) and specific cis-regulatory elements in target transcripts is the basis for most posttranscriptional regulation of gene expression (Figure 1; Moore, 2005).

Although there are a variety of cis-regulatory elements, for example, the cytoplasmic polyadenylated element (CPE) and the iron responsive element (IRE) (Charlesworth et al., 2013; Theil, 2015), we focus here on the AU-rich element (ARE), one important cis-element for RNA regulation, which is typically found in the mRNA 3′ untranslated region (UTR). AREs are contained in 5–8% of human mRNAs coding factors involved in various biological functions such as proliferation, differentiation, signal transduction, apoptosis, and metabolism (Barreau et al., 2005; Bakheet et al., 2006). Originally identified as a sequence inducing mRNA decay (Chen and Shyu, 1995), the ARE was subsequently found to be more broadly involved in RNA processing, transport, and translation (Garcia-Maurino et al., 2017).

Many ARE-binding proteins (ARE-BPs) have been identified that bind to this element and mediate its function in posttranscriptional control (Table 1). Most ARE-BPs characterized to date recognize specific AREs in target mRNAs *via* canonical RNA-binding domains (RBDs), for example, the RNA recognition motif (RRM), CCCH tandem zinc finger domain, and KH domain (Hall, 2005; Clery et al., 2008; Nicastro et al., 2015). However, recently developed techniques have identified many new *bona fide* RBPs and revealed the surprising finding that about half of them do not have a conventional RBD (Castello et al., 2012; Beckmann et al., 2015, 2016). Intriguingly, these noncanonical RBPs include several ARE-BPs and analysis of their potentially contributions to ARE function is underway (Garcin, 2018).

**Table 1:** The RBDs, targets, and functions of ARE-BPs in this review.

The features of ARE-binding proteins				
	RNA-binding domain	mRNA stabilization	mRNA destabilization	Other functions
AUF1	Four RRMs	c-fos, c-myc (Brewer, 1991)	TNF-α, IL-1β (Lu et al., 2006b); c-fos (Brewer, 1991); c-myc (Loflin et al., 1999); GM-CSF? (Pioli et al., 2002)	Splicing (Fragkouli et al., 2017); translational repression (Fellows et al., 2013); viral replication (Friedrich et al., 2014)
TTP	Tandem zinc finger domains		TNF-α (Lai and Blackshear, 2001; Su et al., 2012); GM-CSF (Lai et al., 1999; Su et al., 2012); TTP (Brooks et al., 2004; Tchen et al., 2004); IL-10 (Su et al., 2012)	Translational repression (Tao and Gao, 2015; Fu et al., 2016)
ZFP36L1	Tandem zinc finger domains		Dll4 (Desroches-Castan et al., 2011); CDK6 (Chen et al., 2015)	
ZFP36L2	Tandem zinc finger domains		LHR (Ball et al., 2017); H3K4, H3K9 (Dumdie et al., 2018)	
KSRP	Four KH domains		Myogenin (Giovarelli et al., 2014)	Viral translation repression (Lin et al., 2009); miRNA maturation (Ruggiero et al., 2009; Trabucchi et al., 2009); splicing (Min et al., 1997)
HuR	Three RRMs	c-fos, cox2, TNF-α (Katsanou et al., 2005); SIRT1 (Calvanese et al., 2010)		Translational control in neocortex (Kraushar et al., 2014)
Nuronal Hu proteins	Three RRMs	GAP-43 (Mobarak et al., 2000); APP, BACE1 (Kang et al., 2014)	HuR (Mansfield and Keene, 2012)	Splicing (Fragkouli et al., 2017); translation stimulation (Fukao et al., 2009); miRNA attenuation (Fukao et al., 2014)
GAPDH	Rossmann fold	CSF-1 (Zhou et al., 2008)	Cox-2 (Ikeda et al., 2012); ET-1 (Rodriguez-Pascual et al., 2008)	Translational repression (Chang et al., 2013)
LDHM	Rossmann fold		GM-CSF? (Pioli et al., 2002)	Interaction with AUF1 (Pioli et al., 2002)

Blue colors show canonical ARE-BPs, and red color shows noncanonical ARE-BPs.

A common characteristic of many ARE-BPs is that they shuttle between nucleus and cytoplasm, but they exhibit different functions depending on their localization to control gene expression (Gama-Carvalho and Carmo-Fonseca, 2001). ARE-BP localization and function are both tightly regulated by posttranslational modifications (PTMs) and interactions with other factors (Chen and Shyu, 2014; Shen and Malter, 2015).

In this review, we summarize (1) the function of ARE-BPs to control mRNA stability or translation in the cytoplasm and RNA processing in the nucleus, (2) the biological and pathological importance of gene regulation by ARE-BPs, and (3) the regulation of ARE-BP function, particularly through PTM.

## mRNA Stability and Translational Control by ARE-BPs in the Cytoplasm

AUF1, also known as heterogeneous nuclear ribonucleoprotein D (hnRNP D), was the first identified ARE-BP that can destabilize mRNA (Brewer, 1991). AUF1-KO mice exhibit symptoms of severe endotoxic shock due to excessive production of tumor necrosis factor-α (TNF-α) and interleukin-1β (IL-1β), which results from failure to degrade these mRNAs (Lu et al., 2006b). AUF1 was also found to destabilize mRNAs encoding c-fos and c-myc (Brewer, 1991; Loflin et al., 1999), although another study reported that AUF1 stabilizes these mRNAs (Xu et al., 2001). These apparently conflicting results suggest that the function of AUF1 is not fixed, but can be differentially regulated depending on the cell type and specific conditions (Gouble et al., 2002). AUF1 forms the AUF1- and signal transduction-regulated complex (ASTRC) with several factors [eIF4G, poly(A)-binding protein (PABP) C1, Hsp27, and Hsp70] (Laroia et al., 1999; Lu et al., 2006a; Sinsimer et al., 2008). This complex is required for AUF1-mediated mRNA decay, but its molecular mechanism of action is still unknown.

**Figure 2:**
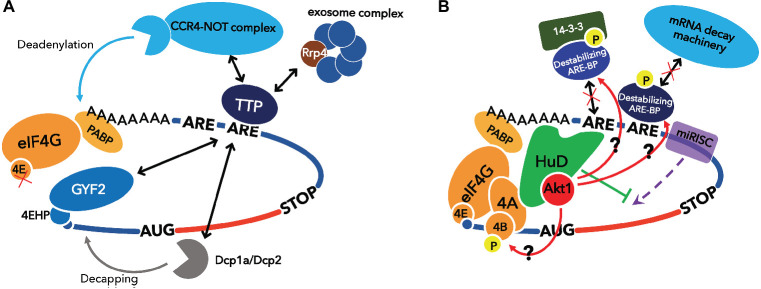
Functional model of destabilizing ARE-BP, TTP and stabilizing ARE-BP, HuD. **(A)** TTP induces mRNA decay by recruiting CCR4-NOT complex, exosome complex, and Dcp1a/Dcp2 complex and represses translation by recruiting 4EHP *via* binding GYF2. **(B)** HuD stimulates translation *via* direct binding to eIF4A and the poly(A) tail. miRISC represses translation by dissociation of eIF4A from the translation initiation complex, and this inhibitory effect on translation initiation is attenuated by HuD. HuD also binds Akt/PKB, which phosphorylates destabilizing ARE-BPs such as KSRP, TTP, ZFP36L1, and ZFP36L2 to inactivate them, and eIF4B to stimulate helicase activity of eIF4A.

TTP is a destabilizing ARE-BP with a well-characterized molecular mechanism. This protein has a tandem zinc finger RBD and binds the 3′UTR of mRNAs coding TNF-α and granulocyte macrophage colony-stimulating factor (GM-CSF), and induces mRNA decay (Lai et al., 1999; Lai and Blackshear, 2001). The mRNA coding for TTP also contains AREs in its 3′UTR, and thus, TTP regulates its own expression levels by a negative feedback (Brooks et al., 2004; Tchen et al., 2004). TTP recruits the CCR4-NOT complex to target mRNAs *via* direct binding to its subunits, CNOT1 and CNOT9 (Fabian et al., 2013; Bulbrook et al., 2018). TTP also interacts with the Dcp1a/Dcp2 complex involved in decapping and a component of the exosome, Rrp4, to degrade mRNA (Lykke-Andersen and Wagner, 2005). Furthermore, TTP represses translation by recruitment of 4EHP to target mRNAs through interaction between its PPPPG motif and GYF2 (Figure 2A; Tao and Gao, 2015; Fu et al., 2016). 4EHP has affinity for the 5′-end cap structure like eIF4E, but does not bind eIF4G. Therefore, 4EHP represses translation by competing with eIF4E for the cap (Morita et al., 2012). The TIS11 family, to which TTP belongs, also contains two other members, ZFP36L1 and ZFP36L2. Although these factors differ from each other in their tissue distribution and target mRNAs, they have about 70% homology, including the CNOT1 binding site, and both induce mRNA decay (Sanduja et al., 2011).

K-homology splicing regulatory protein (KSRP) was initially identified as a nuclear factor involved in transcription and splicing (Davis-Smyth et al., 1996; Min et al., 1997). Subsequently, it was reported that KSRP binds the ARE using two of four KH domains, KH3 and KH4 (Gherzi et al., 2004), and destabilizes target mRNAs by recruitment of poly(A)-specific ribonuclease (PARN) and exosome to mRNAs (Chen et al., 2001; Chou et al., 2006). Furthermore, it was shown that KSRP interacts with the enterovirus 71 internal ribosomal entry site (IRES) and behaves as an IRES trans-acting factor (ITAF) to negatively regulate viral translation (Lin et al., 2009).

Unlike the ARE-BPs introduced so far, Hu proteins are ARE-BPs that stabilize their target mRNAs. The Hu protein family consists of four members. HuR is ubiquitously expressed, whereas HuB, HuC, and HuD are mainly expressed in neurons. All members of Hu proteins have three RRMs. RRM1 and RRM2 recognize ARE, and RRM3 binds the poly(A) tail (Ma et al., 1997). HuR binds to the ARE in the mRNAs encoding c-fos, Cox 2, and TNF-α in competition with TTP or KSRP and stabilizes these mRNAs (Fan and Steitz, 1998b; Katsanou et al., 2005). Furthermore, HuR associates with eIF2 alpha kinase 4 and may temporally define translation in the developing neocortex (Kraushar et al., 2014). Neuronal Hu proteins are thought to regulate and induce neuronal differentiation through stabilizing target mRNAs (Okano and Darnell, 1997; Mobarak et al., 2000; Akamatsu et al., 2005). Fukao et al. previously showed that HuD stimulates translation initiation *via* direct binding to the poly(A) tail and eIF4A (Fukao et al., 2009). Furthermore, Fujiwara et al. demonstrated that physical interaction between HuD and the active form of Akt/PKB is required for morphological alterations such as neurite outgrowth in PC12 cells undergoing a neuronal differentiation program (Fujiwara et al., 2012). Akt/PKB directly phosphorylates eIF4B, whose phosphorylation stimulates the RNA helicase activity of eIF4A (Rozen et al., 1990; Altmann et al., 1993; van Gorp et al., 2009). Thus, it is possible that HuD recruits Akt/PKB to the translation initiation complex to stimulate eIF4A activity on its ARE-containing mRNAs (Figure 2B).

## Nuclear Function of ARE-BPs

Regulation of mRNA stability, localization, and translation is a cytoplasmic function of ARE-BPs, yet most ARE-BPs shuttle between nucleus and cytoplasm, thereby suggesting that these proteins also have nuclear functions. Indeed, several nuclear functions for ARE-BPs have been identified. For example, in recent years, it was shown that KSRP has a novel nuclear function involved in maturation of a subset of microRNAs (Ruggiero et al., 2009; Trabucchi et al., 2009). KSRP binds to a terminal loop of miRNA precursors and promotes both steps of biogenesis: conversion of pri-miRNAs to pre-miRNAs in the nucleus by Drosha and pre-miRNA processing to mature miRNAs in the cytoplasm by Dicer (Trabucchi et al., 2009).

Hu proteins have a domain regulating nuclear-cytoplasmic shuttling located in a linker region between RRM2 and RRM3 (Fan and Steitz, 1998a; Kasashima et al., 1999). A recent study showed that AREs are abundant in introns of human genes and that HuR regulates expression of genes containing these intronic AREs (Bakheet et al., 2018). The pre-mRNAs coding for HuR undergo alternative polyadenylation leading to transcript variants with different lengths of 3′UTR and stability (Al-Ahmadi et al., 2009). Because HuR impairs neuronal differentiation by promoting cell proliferation, neuronal Hu proteins decrease HuR expression by binding to the pre-mRNA of HuR at the polyadenylation site to produce a less stable mRNA bearing the long 3′UTR (Mansfield and Keene, 2012). Neuronal Hu proteins are also involved in neuron-specific alternative splicing by utilizing AUF1 as a co-factor (Fragkouli et al., 2017).

TIS11 family proteins have a potential nuclear localization signal within the zinc finger domain (Murata et al., 2002; Phillips et al., 2002; Twyffels et al., 2013). In the nucleus, TTP in association with poly(A)-binding protein nuclear 1 (PABPN1) inhibits poly(A) tail synthesis on mRNAs which contain AREs, such as TNF-α, GM-CSF, and IL-10, thereby promoting degradation of these transcripts (Su et al., 2012). Under hypoxia, ZFP36L1 has been reported to reduce expression level of Delta-like 4 (Dll4) involved in cell fate determination in angiogenesis by inhibiting cleavage at the polyadenylation site of the *Dll4* mRNA (Kume, 2009; Desroches-Castan et al., 2011).

## Noncanonical ARE-BPs

Recently, systematic investigation of RBPs has been performed in various cell types (yeast and cultured cells) by interactome capture assays (Castello et al., 2012; Beckmann et al., 2015, 2016). Protein-RNA interactions are immobilized by conventional UV crosslinking (cCL) by 254 nm UV irradiation or photoactivatable ribonucleoside-enhanced (PAR-) CL by 365 nm UV irradiation using cells by which photoactivatable 4-thiouridine (4 SU) is taken up. Then, mRNA-RBP complexes are captured by oligo(dT) beads, and the proteins are analyzed by mass spectrometry after digestion of mRNAs. As a result, many novel RBPs were detected. Surprisingly, about half of these have no conventional RBD (Castello et al., 2012; Beckmann et al., 2015, 2016). Many well-known metabolic enzymes are among these noncanonical RBPs. A typical example of a metabolic enzyme that has been identified as noncanonical RBP is ACO1/IRP1. When iron levels are in the normal physiological range, ACO1/IRP1 functions as a cytoplasmic aconitase in the TCA cycle. However, in iron-deficient conditions, ACO1/IRP1 behaves as a sequence-specific RBP that recognizes a certain stem-loop structure, the iron-responsive element (IRE) (Constable et al., 1992). ACO1/IRP1 binds the 3′UTR of the mRNA coding transferrin involved in iron uptake and stabilizes this mRNA (Casey et al., 1988; Mullner and Kuhn, 1988). It also binds to an IRE in the 5′UTR of the mRNA encoding ferritin, a protein involved in iron storage. In this case, it inhibits translation (Hentze et al., 1987), thereby regulating the intracellular iron level. This classic example of a metabolic enzyme moonlighting as an RBP illustrates how cellular metabolic states can be intimately connected with posttranscriptional regulation of gene expression (Castello et al., 2015).

Further evidence to support this principle is found in the glycolytic enzyme, glyceraldehyde-3-phosphate dehydrogenase (GAPDH), which is a noncanonical ARE-BP (Nagy and Rigby, 1995). GAPDH binds the ARE *via* a Rossmann fold which binds NAD^+^/NADH, and thus, NAD^+^ abundance affects binding activity of GAPDH to the ARE (Nagy and Rigby, 1995; Rodriguez-Pascual et al., 2008; Ikeda et al., 2012). Indeed, a switch from oxidative phosphorylation to aerobic glycolysis when T lymphocytes are activated promotes dissociation of GAPDH from the ARE in the mRNA coding for interferon-γ (IFN-γ) and increases expression of IFN-γ (Chang et al., 2013). GAPDH also binds to mRNAs containing AREs, such as those encoding colony stimulating factor-1 (CSF-1), cyclooxygenase-2 (Cox-2), and endothelin-1 (ET-1), and regulates stability or translation of these mRNAs (Rodriguez-Pascual et al., 2008; Zhou et al., 2008; Ikeda et al., 2012). Likewise, lactate dehydrogenase (LDH) M, which is a glycolytic enzyme, also binds an ARE in the mRNA coding for GM-CSF by a Rossmann fold in an NAD^+^ concentration-dependent manner (Pioli et al., 2002). Moreover, LDHM directly interacts with AUF1. This interaction is thought to complement low binding specificity of AUF1, which also binds various RNAs even without AREs (Kiledjian et al., 1997; Eversole and Maizels, 2000), and to be utilized for recruitment of AUF1 to target mRNAs (Pioli et al., 2002).

## Biological Functions of ARE-BPs in Health and Disease

The fact that AREs are found mainly in mRNAs coding for inflammatory cytokines and growth factors suggests the potential for coordinated regulation of specific biological processes by ARE-BPs (Barreau et al., 2005; Khabar, 2017; Turner and Diaz-Munoz, 2018).

ZFP36L1 and ZFP36L2 have redundant functions in T-cell and B-cell maturation (Hodson et al., 2010; Galloway et al., 2016). During T-cell maturation, ZFP36L1 and ZFP36L2 limit the cell cycle and repress the DNA damage response induced by double-strand DNA breaks (Vogel et al., 2016). Moreover, ZFP36L1 promotes monocyte/macrophage differentiation by controlling mRNA stability of CDK6 (Chen et al., 2015). It was reported that mice that lack the N-terminal 29 amino acids of ZFP36L2 are infertile (Ramos et al., 2004; Ramos, 2012), due to failure to control expression of luteinizing hormone receptor (LHR) by ZFP36L2 (Ball et al., 2014). More recently, oocyte-specific KO of ZFP36L2 in mice showed that this protein controls expression of histone demethylases targeting H3K4 and H3K9 and induces global transcriptional silencing in the oocyte, which is important for the oocyte-to-embryo transition (Dumdie et al., 2018).

Neuronal Hu proteins are involved in alternative splicing of amyloid precursor protein (APP) (Fragkouli et al., 2017). The APP gene contains 18 exons and 3 isoforms: APP770 contains all exons, APP751 lacks exon 8, and APP695 lacks exons 7 and 8. In the brain of Alzheimer’s disease patients, APP695 is decreased, whereas APP770 is increased (Moir et al., 1998; Matsui et al., 2007). Neuronal Hu proteins promote expression of APP695 instead of APP770 (Fragkouli et al., 2017). On the other hand, HuD stabilizes the mRNAs for APP, as well as β-site APP-cleaving enzyme 1 (BACE1), which induces processing from APP to amyloid-β (Kang et al., 2014).

ARE-BPs are also implicated in other neurological disorders. A human genetics study identified TIA1 mutations in amyotrophic lateral sclerosis (ALS) and frontotemporal dementia (FTD) patients (Mackenzie et al., 2017). Interestingly, this same study showed that these mutations promote phase separation of TIA1 protein and affect the dynamics of stress granules, which are themselves suggested to be important in ALS pathology (Li et al., 2013; Zhang et al., 2018).

HuD may also be involved in ALS and another neurological disorder, spinal muscular atrophy (SMA). Direct evidence for a contribution of HuD to ALS is lacking, but it was found to form insoluble aggregates in the cytoplasm with TDP-43, an RBP heavily implicated in ALS and FTD (Fallini et al., 2011), thereby raising the possibility of pathological interactions between these two RBPs. In addition to nuclear functions in pre-mRNA processing, TDP-43 also represses translation of specific mRNAs in *Drosophila* ALS models and cultured mammalian cells (Majumder et al., 2012, 2016; Coyne et al., 2014, 2017), although the exact connection between these mRNAs and ALS remains unclear. More recently, TDP-43 was also shown to function as an mRNA-specific translational enhancer for the mRNAs encoding CAMTA1 and DENND4, both of which are directly linked to ALS and neurodegenerative disease (Neelagandan et al., 2019). Whether HuD contributes to this regulation remains to be determined. However, the *Camta1* and *Dennd4a* mRNAs both contain many AREs based on *in silico* analyses (Fallmann et al., 2016). This observation, taken together with HuD’s ability to function as an mRNA-specific translational enhancer *via* AREs (Fukao et al., 2009), raises the possibility that HuD might potentially function as a co-factor in TDP-43-driven translational enhancement of *Camta1* and *Dennd4a* mRNAs.

SMA is caused by lack or mutation of survival of motor neuron protein (SMN) (Burghes and Beattie, 2009). SMN interacts with HuD on mRNAs such as the one coding for candidate plasticity-related gene 15 (cpg15), and forms an RNA granule (Akten et al., 2011; Fallini et al., 2011; Hubers et al., 2011). The tudor domain of SMN is important for interaction between SMN and HuD, and an SMN mutant from severe SMA patients bearing a mutation in the Tudor domain cannot interact with HuD (Buhler et al., 1999; Fallini et al., 2011; Hubers et al., 2011). What does this interaction between HuD and SMN mean? We previously reported translation stimulation by HuD (Fukao et al., 2009). Another group showed that SMN represses translation of certain mRNAs, and the Tudor domain mutant of SMN is not able to repress translation (Sanchez et al., 2013). Repression of ectopic translation and induction of translation initiation in response to local stimulatory cues are important components of local translation in neuronal compartments. Moreover, SMN is closely involved in axonal translation (Bernabo et al., 2017). Therefore, an interesting possibility is that SMN and HuD could have opposite, but complementary, roles in the context of neuronal mRNP transport and translation. According to this view, SMN could function as a brake to suppress ectopic translation while mRNPs are transported to sites where local protein synthesis would occur. Conversely, HuD’s role would be to promote translation initiation at these sites in response to local neuronal cues. Future studies in primary neuronal cultures could examine this possibility.

## Regulation of ARE-BP Function

As can be seen from the examples of SMN and HuD, functional regulation of ARE-BPs is strongly related to biological functions and diseases. Thus, function of ARE-BPs is controlled by several factors such as long noncoding (lnc) RNA, other proteins, and PTMs.

H19 is an lncRNA expressed in embryo and skeletal muscle (Bartolomei et al., 1991). A recent study showed that H19 directly binds KSRP and promotes destabilization of the mRNA for myogenin by KSRP, thus favoring myogenic differentiation (Giovarelli et al., 2014). Overexpressed in colon carcinoma-1 (OCC-1), an lncRNA binds HuR and enhances binding of the β-TrCP1 E3-ubiquitin ligase, thereby promoting destabilization of the HuR protein (Pibouin et al., 2002; Lan et al., 2018).

Arginine methylation is a common feature of a large population of RGG box proteins, which are involved in many aspects of mRNA metabolism (Rajyaguru and Parker, 2012). In some cases, arginine methylation has been shown to regulate the function of ARE-BPs containing RGG boxes. For example, HuR is methylated at R217 by coactivator-associated arginine methyltransferase 1 (CARM1) and methylated HuR binds the mRNA encoding the histone deacetylase, Sirtuin 1 (SIRT1) to stabilize it (Calvanese et al., 2010). Many hnRNP proteins that contain RGG boxes are also subject to arginine methylation, thereby potentially affecting their localization and RNA-binding activity (Yu, 2011). However, while it was reported that hnRNP D/AUF1 is methylated, this did not seem to affect either localization or RNA-binding activity of AUF1 (DeMaria et al., 1997; Sarkar et al., 2003). Recently, it was shown that arginine methylation of AUF1 is involved in translational repression of the mRNA coding for vascular endothelial growth factor (VEGF) (Fellows et al., 2013). Furthermore, this PTM also affects AUF1’s role as a host factor during the replication of the West Nile virus genome (Friedrich et al., 2014) In this case, arginine methylation of AUF1 by protein arginine N-methyltransferase 1 (PRMT1) promotes AUF1 function as an RNA chaperone (Friedrich et al., 2016).

Many ARE-BPs are phosphorylated. In some cases, the regulatory effects of phosphorylation, as well as the signaling pathways and kinases responsible, have been determined. For example, KSRP has two independent phosphorylation sites in its C-terminal and KH1 domains (Briata et al., 2005; Diaz-Moreno et al., 2009). The C-terminal Thr692 of KSRP is phosphorylated by p38/MAPK to promote destabilization of target mRNAs (Briata et al., 2005). On the other hand, Ser193 in the KH1 domain is phosphorylated by Akt/PKB to localize KSRP in the nucleus *via* binding of 14-3-3 proteins, thereby inhibiting mRNA decay in the cytoplasm (Diaz-Moreno et al., 2009). TIS11 family proteins, TTP, and ZFP36L1 and ZFP36L2 are also phosphorylated by p38/MAPK or Akt/PKB and recognized by 14-3-3 proteins (Chrestensen et al., 2004; Schmidlin et al., 2004; Benjamin et al., 2006). Phosphorylation of TTP at Ser52 and Ser178 reduces interaction with the CCR4-NOT complex and thereby upregulates mRNA stability (Marchese et al., 2010). Conversely, phosphorylation at Ser334 of ZFP36L1 also decreases interaction with the CCR4-NOT complex, but increases affinity to Dcp1a to promote mRNA decay (Rataj et al., 2016).

HuD is subject to phosphorylation by PKC to promote its mRNA stabilizing activity (Lim and Alkon, 2012). On the other hand, we previously demonstrated that HuD interacts with Akt/PKB, although Akt/PKB does not lead to HuD phosphorylation (Fujiwara et al., 2012). This interaction might recruit Akt/PKB, which phosphorylates and inactivates destabilizing ARE-BPs such as KSRP and TIS11 family proteins, to ARE-containing mRNAs. This suggests that HuD can not only compete with destabilizing ARE-BPs but also potentially inactivate them on the same mRNA through phosphorylation by Akt/PKB to stabilize mRNA. We also showed that HuD attenuates translational repression by the miRNA-induced silencing complex (miRISC), which leads to mRNA decay as well as destabilizing ARE-BPs (Fukao et al., 2014). These observations support a central role for HuD in stabilizing mRNA and promoting translation (Figure 2B).

## Conclusion and Perspectives

The ARE has been studied for a long time, and about 20 ARE-BPs have been identified since discovery of first ARE-BP, AUF1 (Brewer, 1991; Garcia-Maurino et al., 2017). The specific target mRNAs for different ARE-BPs, as well as their molecular functions on these mRNAs, and contribution of this regulation to specific biological processes are gradually being uncovered. However, with a few exceptions, the molecular mechanisms used by ARE-BPs to regulate their targets are still unknown. In particular, the mechanism to recognize and control specific targets from the large number of transcripts that have AREs is an open question. Recently, Ball et al. revealed that ZFP36L2, but not ZFP36L1, recognizes one of three AREs in 3′UTR of mRNA coding LHR, and this ARE is located within a hairpin structure (Ball et al., 2014, 2017). This indicates that not only the ARE sequence but also proximal RNA secondary structure affects the binding specificity of ARE-BPs. Future experimental and *in silico* approaches to understand the determinants of ARE recognition by specific ARE-BPs’ analysis will thus be needed to incorporate RNA structure, as well as sequence. Moreover, as shown in the example of LDHM and AUF1 (Pioli et al., 2002), it will also be necessary to study the influence of the interaction between ARE-BPs on specific ARE recognition and molecular regulatory mechanisms on the same transcripts. Finally, systematic studies have shown that the relative spacing of 3′UTR cis-elements and associated regulatory proteins can have strong contextual effects on regulation (Pique et al., 2008). Thus, to understand fully ARE-BP function and mechanism, it will be important to examine interplay between AREs, ARE-BPs, and other neighboring cis-elements within specific 3′UTRs.

## Author Contributions

HO, AF, YF, KD, and TF wrote and discussed the article.

### Conflict of Interest Statement

The authors declare that the research was conducted in the absence of any commercial or financial relationships that could be construed as a potential conflict of interest.
