# Corrigendum: Emerging Evidence of Translational Control by AU-Rich Element-Binding Proteins

**DOI:** 10.3389/fgene.2021.715196

**Published:** 2021-06-28

**Authors:** Hiroshi Otsuka, Akira Fukao, Yoshinori Funakami, Kent E. Duncan, Toshinobu Fujiwara

**Affiliations:** ^1^Graduate School of Frontier Sciences, University of Tokyo, Kashiwa, Japan; ^2^Kindai University, Higashi-osaka, Japan; ^3^University Medical Center Hamburg-Eppendorf, Hamburg, Germany

**Keywords:** RNA-binding proteins, AU-rich element, ARE-binding proteins, translational control, mRNA decay

In the original article, the reference for LHR (4th column, 5th row in Table 1) was incorrectly written as (Chen et al., 2015). It should be (Ball et al., 2017). Corrected Table 1 is given below. The authors apologize for this error and state that this does not change the scientific conclusions of the article in any way. The original article has been updated.

**Table 1:**
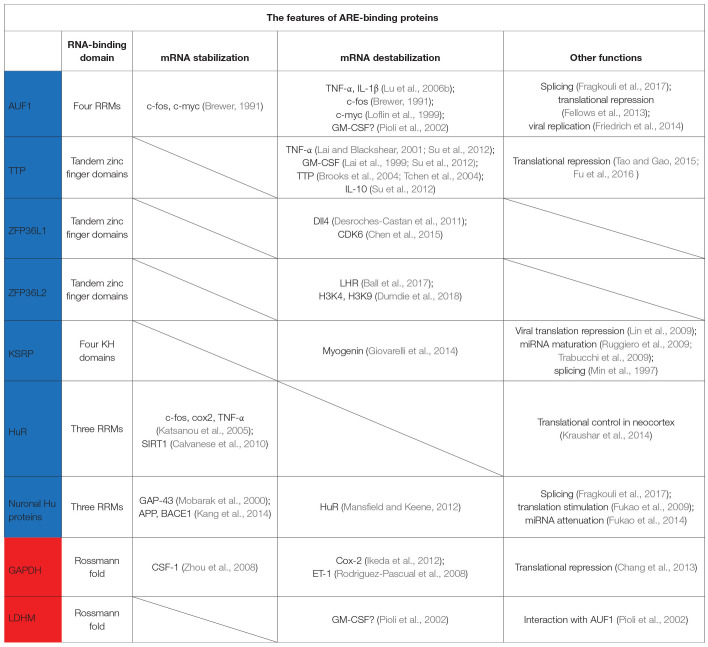
The RBDs, targets, and functions of ARE-BPs in this review.

*Blue colors show canonical ARE-BPs, and red color shows noncanonical ARE-BPs*.
